# Progress of infant obesity is associated with higher aspartyl-glutamate and alanyl-aspartate in maternal and neonatal blood

**DOI:** 10.3389/fpubh.2025.1623604

**Published:** 2025-08-14

**Authors:** Yuanying Liu, Mingyue Ma, Yanhong Yu, Chunyuan Yang, Jianling Yang, Tongyan Han, Yongqing Wang

**Affiliations:** ^1^National Clinical Research Center for Obstetrics and Gynecology, Department of Obstetrics and Gynaecology, Peking University Third Hospital, Beijing, China; ^2^Department of Community Health Sciences, UIC School of Public Health, Chicago, IL, United States; ^3^Department of Pediatric, Peking University Third Hospital, Beijing, China; ^4^Center of Basic Medical Research, Institute of Medical Innovation and Research, Peking University Third Hospital, Beijing, China

**Keywords:** infants, glucose and lipid metabolism, obesity, metabolomics, pregnancy

## Abstract

**Objective:**

Neonatal obesity may be associated with the intra-uterine environment during pregnancy. The objective of this study was to evaluate the risk of neonatal obesity born from the mothers with abnormal glucose and lipid metabolism.

**Methods:**

Twenty neonates born from maternal glucose and lipid metabolism disorders and developed obesity at 6 months of age were enrolled as study group, and 20 neonates without obesity were included as control group. Non-targeted metabolomic analysis was performed in maternal serum during pregnancy and neonatal cord blood at birth to identify differential metabolites.

**Results:**

The concentrations of aspartyl-glutamate and alanyl-aspartate in maternal serum progressively rise steadily as gestational age advances, peaking in umbilical cord blood. Additionally, at each stage of pregnancy (early, middle, and late), the levels in both maternal serum and umbilical cord blood are significantly higher in the obese group than in the non-obese group. Their mechanisms of action may be associated with pathways involving immune-inflammatory regulation, energy metabolism, and gut microbiota modulation. Their mechanisms of action may be associated with pathways involving immune-inflammatory regulation, energy metabolism, and gut microbiota modulation.

**Conclusion:**

Through the analysis of maternal blood during pregnancy and umbilical cord blood, this study putatively identified some differential metabolites associated with neonatal obesity. In the future, it is expected that analyzing maternal blood or umbilical cord blood at birth could help predict potential infant obesity risks, enabling more dietary guidance and interventions during infancy to reduce the risk of obesity later in life.

## Introduction

Research indicates that abnormalities in glucose and lipid metabolism during the neonatal period are closely associated with the development of obesity later in life. Such metabolic disturbances may already exist in embryonic period and exert profound impacts on individual health (1). The study on the Hyperglycemia and Adverse Pregnancy Outcome (HAPO) cohort shows that exposure to higher levels in utero is significantly associated with childhood glucose and insulin resistance. In recent years, with changes in lifestyle and nutritional structure, an increasing number of neonates are influenced by intrauterine environments, leading to potential risks of glucose and lipid metabolism abnormalities at birth. This population exhibits an elevated risk of developing neonatal obesity in the long term. Obesity not only increases the risk of chronic diseases such as cardiovascular diseases, type 2 diabetes, and metabolic syndrome but may also have long-term adverse effects on children’s growth, psychological well-being, and quality of life (2). Early prediction of neonatal obesity risk associated with glucose and lipid metabolism abnormalities is crucial for developing early interventions and improving long-term health outcomes (3).

However, current research on the association between neonatal glucose and lipid metabolism abnormalities and obesity is largely confined to traditional biochemical indicators, lacking comprehensive analysis of metabolic pathways (4). Untargeted metabolomics, as a systems biology approach, enables a holistic and unbiased analysis of dynamic changes in metabolites within biological systems, providing new perspectives for elucidating the underlying mechanisms linking metabolic abnormalities and obesity. Based on this, the present study aims to utilize untargeted metabolomics technology to identify biomarkers capable of predicting the risk of obesity in neonates with potential glucose and lipid metabolism abnormalities, thereby offering scientific evidence for early risk assessment and personalized intervention. Through this research, we aim to pioneer new approaches for the early prevention and control of neonatal obesity and provide novel insights into the mechanisms of related metabolic diseases.

## Method

This study is a prospective case–control study based on a longitudinal pregnancy cohort. It includes neonates with potential risks of glucose and lipid metabolism abnormalities, whose mothers underwent regular prenatal care and delivered at Peking University Third Hospital from April 2023 to June 2024. Follow-up was conducted at 6 months postpartum to monitor the newborns’ body length, weight, and growth development. The diagnostic criteria for obesity were defined as a body weight greater than 9.65 kg for male infants and greater than 9.2 kg for female infants at 6 months postpartum. A total of 20 obese newborns were selected as the study group, and 20 non-obese newborns born during the same period were selected as the control group. The study was conducted in accordance with the Declaration of Helsinki and approved by the Ethics Committee of Peking University Third Hospital, and written informed consent was obtained from the parents of all enrolled newborns, The study was registered as a clinical trial at the Chinese Clinical Trial Registry on September 10, 2024(registration number: ChiCTR240008953).

Inclusion Criteria including Preterm infants; Low birth weight infants; Macrosomia; Mothers diagnosed with diabetes before pregnancy; Mothers with glucose metabolism abnormalities such as impaired glucose tolerance.

Exclusion Criteria including newborns with confirmed malformations or chromosomal abnormalities; newborns diagnosed with endocrine diseases or congenital genetic metabolic disorders.

Mothers of the newborns underwent regular prenatal examinations at our hospital. Venous blood samples were collected using EDTA anticoagulant tubes during the following gestational periods: Early pregnancy (5–12 weeks of gestation); Mid-pregnancy (22–26 weeks of gestation); Late pregnancy (28–32 weeks of gestation). Additionally, umbilical cord venous blood was collected at delivery. All specimens were centrifuged at 1000 r/min for 15 min at 2–8°C within 30 min of collection. The supernatant was separated and stored at −80°C for subsequent analysis, with precautions taken to avoid repeated freeze–thaw cycles. Only cases with complete blood samples from early, mid, and late pregnancy along with umbilical cord blood samples were included in the study.

After mixing 10 μL from each actual sample, QC samples are obtained. A QC sample is added before and after the formal sample in the analysis queue, and one QC sample is added every 10 samples in between. The ionization signals in the QC samples are monitored based on the peak intensity of the internal standard to ensure that there is no decline in signal intensity (within 20%) and that the retention times show no drift (within 0.05 min) throughout the entire run. The average QC correlation coefficient is 0.99, indicating that the metabolite data generated from liquid chromatography-mass spectrometry analysis is highly consistent, reflecting high data quality.

Untargeted metabolomics was conducted at LipidALL Technologies. Polar metabolites were extracted from 50 μL of plasma using 200 μL of ice-cold methanol containing 0.28 mM phenylhydrazine. Samples were vortexed and kept at −20°C for 1 h for derivatization of alpha-keto acids (5). Following derivatization, samples were centrifuged for 10 min at 12000 rpm at 4°C. Clean supernatant was transferred to a new tube and dried in a SpeedVac under H2O mode. The dried extract was reconstituted in 5% acetonitrile in water prior to LC–MS analysis on an Agilent 1,290 II UPLC coupled to Sciex 5,600 + quadrupole-TOF MS. For reverse phase liquid chromatography (RPLC), polar metabolites were separated on a Waters ACQUITY HSS-T3 column (3.0 × 100 mm, 1.8 μm), while a Waters ACQUITY BEH Amide column (2.1 × 100 mm, 1.7 μm) was utilized for hydrophilic interaction liquid chromatography (HILIC). MS parameters for detection were: ESI source voltage positive ion mode 5.5 k V, negative ion mode −4.5 kV; vaporizer temperature, 500°C; drying gas (N2) pressure, 50 psi; nebulizer gas (N2) pressure, 50 psi; curtain gas (N2) pressure, 35 psi; The scan ranges were set at m/z 60–700 during RPLC, and m/z 70–850 during HILIC analysis, respectively (6). Information-dependent acquisition mode was used for MS/MS analyses of the metabolites. Collision energy was set at (±) 35 ± 15 eV. Data acquisition and processing were performed using Analyst^®^ TF 1.7.1 Software (AB Sciex, Concord, ON, Canada). All detected ions were extracted using MarkerView 1.3 (AB Sciex, Concord, ON, Canada) into Excel in the format of two dimensional matrix, including mass to charge ratio (m/z), retention time, and peak areas, and isotopic peaks were filtered. PeakView 2.2 (AB Sciex, Concord, ON, Canada) was applied to extract MS/MS data and perform comparisons with the Metabolites database (AB Sciex, Concord, ON, Canada), HMDB, and standard references to annotate ion identities (7). A cocktail of isotopically-labeled internal standards (IS) purchased from Cambridge Isotope Laboratories were spiked into the samples for metabolite quantitation, including L-Tryptophan-d5, L-Isoleucine-d10, L-leucine-d10, L-Methionine-d3, L-Valine-d8, L-Proline-d7, L-Alanine-d4, DL-Serine-d3, L-Glutamine-d5, Glycine-13C2, L-Aspartic acid-d3, L-Arginine-13C6, L-Glutamate-d5, L-Lysine-d9, L-Histidine-13C6, Taurine-13C2, Betaine-d11, Urea-(13C,15 N2), L-lactate-d3, Trimethylamine N-oxide-d9, Choline-d13, Malic acid-d3, Citric acid-d4, Succinic acid-d4, Fumaric acid-d2, Hypoxanthine-d3, Xanthine-15 N2, Thymidine (13C10,15 N2), Inosine-15 N4, Cytidine-13C5, Uridine-d2, Methylsuccinic acid-d6, Benzoic acid-d5, Creatine-d3, Creatinine-d3, Glutaric acid-d4, Hippuric acid-d5, Kynurenic acid-d5, L-Citrulline-d4, L-Threonine-(13C4, 15 N), L-Tyrosine-d7, P-cresol sulfate-d7, Sarcosine-d3, Trans-4-hydroxy-L-proline-d3, Uric acid-(13C; 15 N3), Oleic acid-d9, Carnitine-C16:0-d3, Carnitine-C12:0-d9, Carnitine-C14:0-d9, Glycodeoxycholate-d4, L-Carnitine trimethyl-d9, Pyruvate-d3, Pyruvate-d3, vate-d3, L-Asparagine-13C4. Peak areas of endogenous metabolites were normalized to the areas of their corresponding isotopically labeled structural analogues for quantitation. For endogenous metabolites without labeled structural analogues, an automated algorithm selects the optimal internal standard for quantitation based on the rule of minimal coefficients of variations (COVs) after normalization (7).

Normalized metabolite peak areas were log2-transformed, Pareto-scaled, and filtered to retain features detected in ≥ 70% of all tissue samples. Missing-at-random values were imputed within each study group by random forest (missForest 1.5). Metabolites with an absolute log2-fold change ≥ 0.58and Benjamini–Hochberg adjusted *p* < 0.05 were deemed significant. An OPLS-DA model was built in ropls 1.34 using 7-fold cross-validation; variables with VIP > 1.2 and |p(corr)| > 0.4 were regarded as discriminative. Model stability was verified by 2000-time permutation testing (Q^2^-intercept < 0.05). Raw *p*-values from limma were corrected using the Benjamini–Hochberg false-discovery rate (FDR). For pathway enrichment and network topology tests, results were additionally confirmed with the Bonferroni method; only findings passing FDR < 0.05 (or Bonferroni-corrected *p* < 0.05) were interpreted. All analyses were executed in R 4.3.2.

## Result

**Figure 1:**
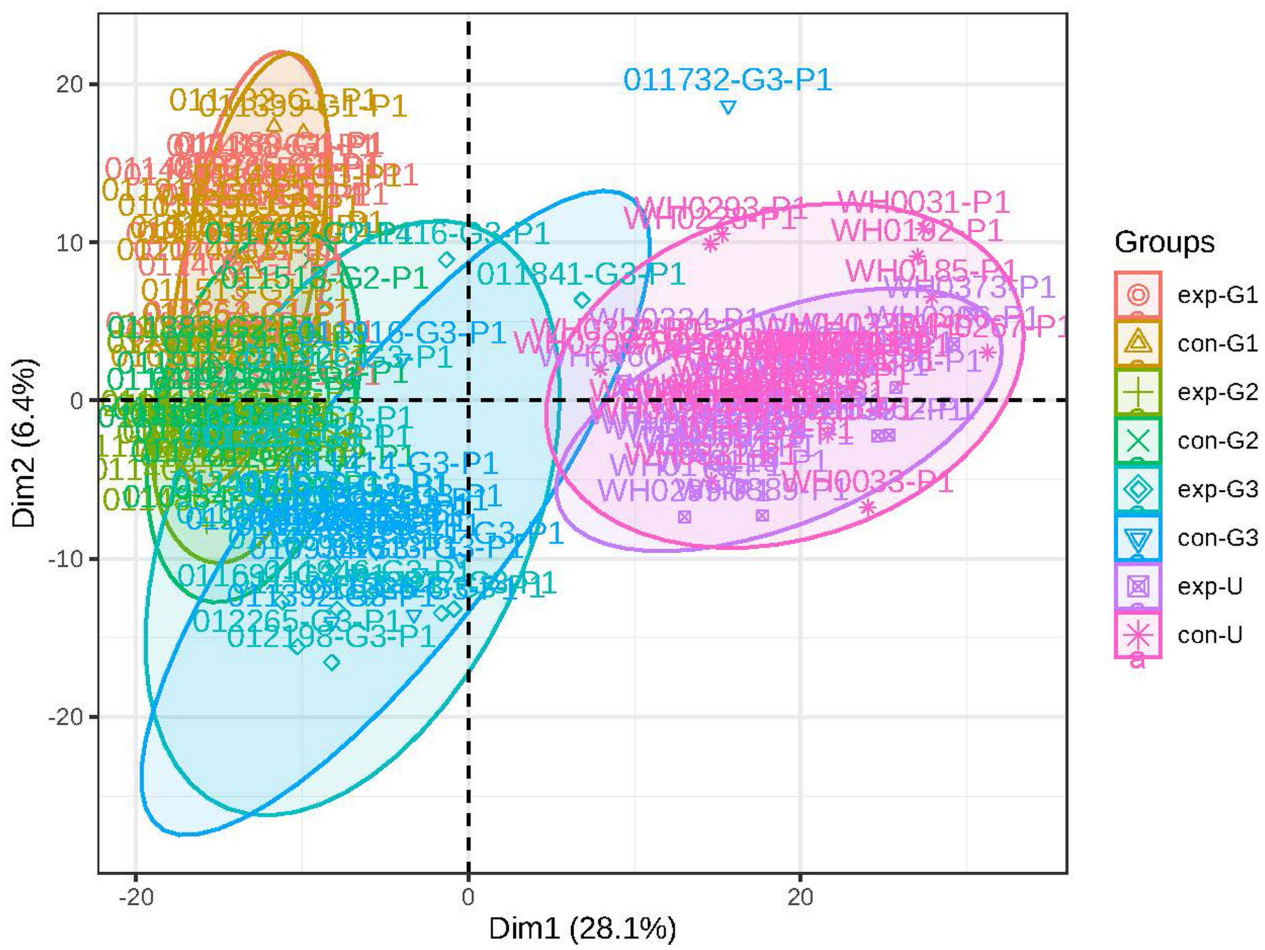
Principal component analysis: the figure shows a discrete trend in the metabolic profiles of different groups (exp represents the obese group, con represents the non-obese group; G1 represents early pregnancy, G2 represents mid-pregnancy, G3 represents late pregnancy, and U represents umbilical blood).

PCA was used to analyze the metabolic profiles of plasma. As shown in Figure 1, within the 95% confidence interval, the samples from each group exhibit clear clustering, indicating good instrument reproducibility, stable sampling methods, and reliable data. In both positive and negative ion modes, the PCA plot can be divided into two parts based on the degree of separation: one cluster corresponds to cord blood, and the other to maternal blood during pregnancy. This suggests significant differences in the metabolite profiles between cord blood and maternal blood, with notable changes in the types or levels of metabolites (see Figure 1).

**Figure 2:**
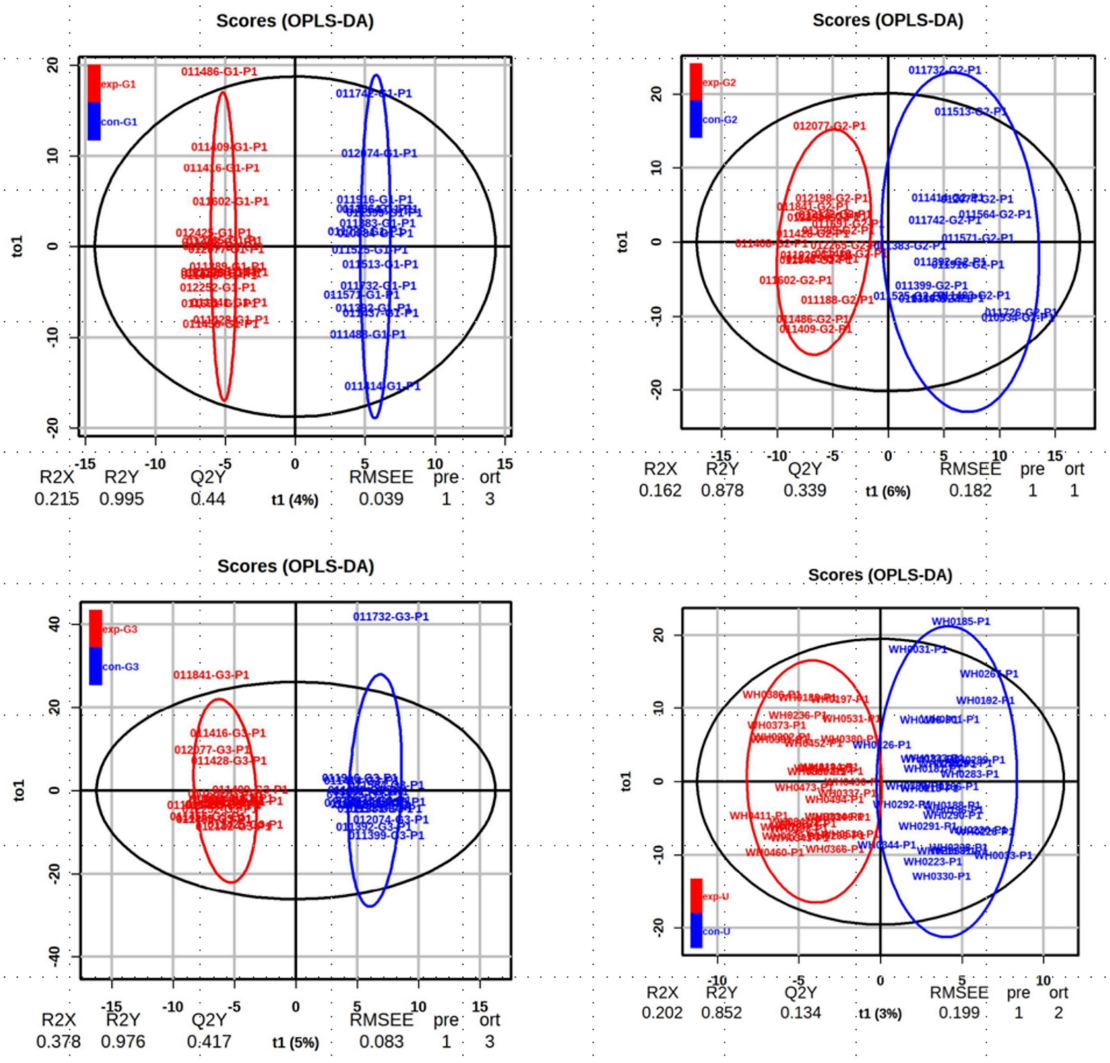
The OPLS-DA score chart: the analysis of obese and non-obese groups at the early, mid, and late stages of pregnancy as well as cord blood.

The OPLS-DA (Orthogonal Partial Least Squares-Discriminant Analysis) was performed on the data from each group, and the results are shown in Figure 2. The analysis indicates that the model has good discriminative ability, with no signs of overfitting.

**Figure 3:**
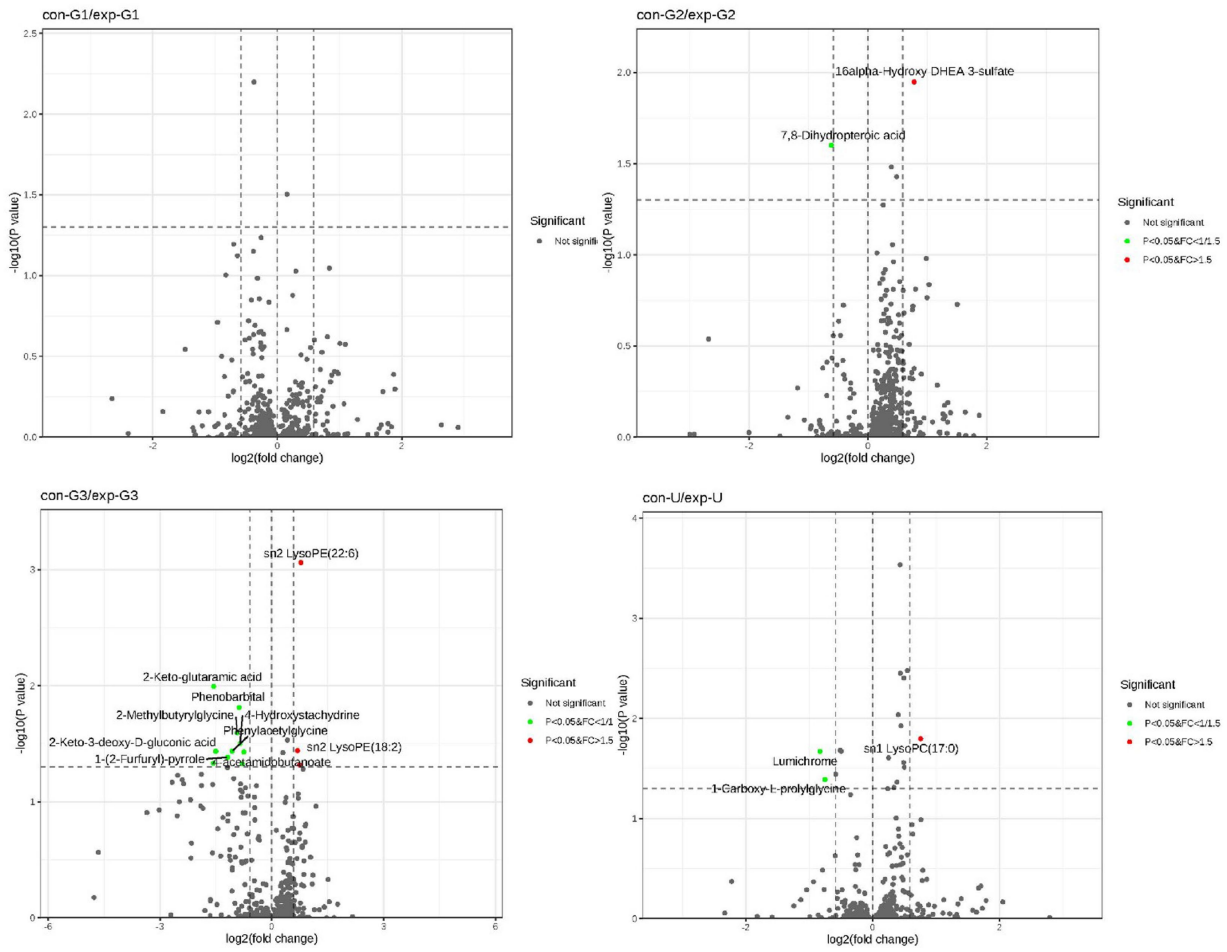
The volcano plot: the figure illustrates the differential metabolites between the two groups at the early, mid, and late stages of pregnancy as well as cord blood.

To identify the differential metabolites between groups, an S-plot was generated, and a volcano plot was constructed by combining the fold change (FC) of metabolites with their statistical significance (*p*-values). This approach allows for a more intuitive visualization of significant differential metabolites. In the volcano plot, metabolites on both sides represent significant upregulation or downregulation, while those at the top are statistically significant, further validating the differences between the groups (as shown in Figure 3).

**Figure 4:**
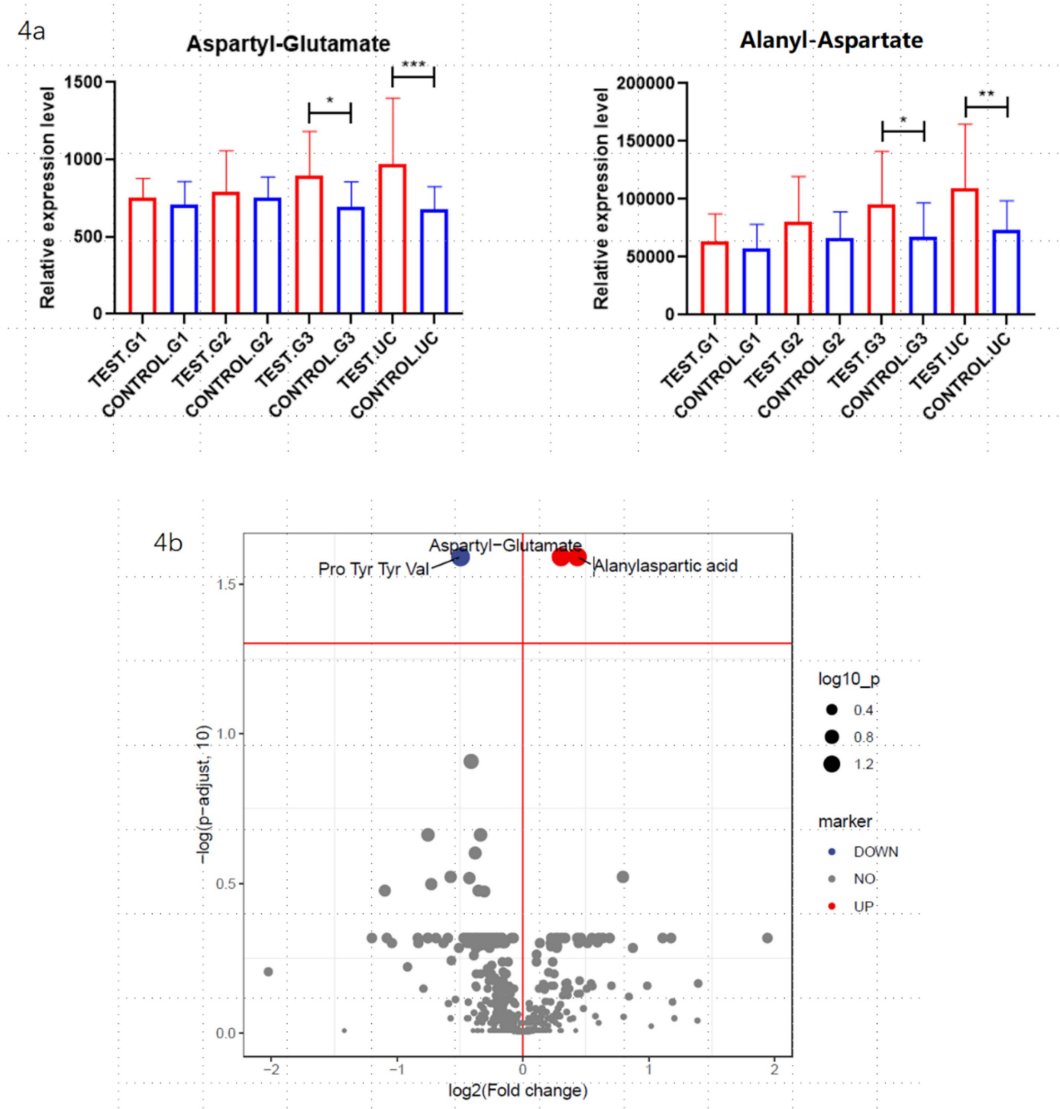
Differential metabolite analysis: the figure shows the differentially expressed metabolites obtained from the overall analysis of the obese and non-obese groups. **(a)** Represents the aspartyl-glutamate and alanyl-aspartate during early, middle and late pregnancy, as well as in cord blood, between the obese and non-obese groups. “*” Indicates that the difference is statistically significant. **(b)** Presents a volcano plot analysis of the differential metabolites.

To identify differential metabolites between the groups, both univariate and multivariate analysis methods were employed. The univariate analysis utilized fold change (FC), while the multivariate analysis was based on the OPLS-DA (Orthogonal Partial Least Squares-Discriminant Analysis) model. Ultimately, two potential biomarkers showing significant differences across the groups were identified: aspartyl-glutamate and alanyl-aspartate. The concentrations of aspartyl-glutamate and alanyl-aspartate in maternal serum progressively increase as gestational age advances, peaking in umbilical cord blood. Their mechanisms of action may be associated with pathways involving immune-inflammatory regulation, energy metabolism, and gut microbiota modulation. These results are illustrated in Figure 4.

Due to significant differences between umbilical cord blood and prenatal blood, we analyzed umbilical cord blood separately and identified 16 significant differential metabolites that are involved in multiple biological processes such as oxidative stress, inflammatory response, and microbiota regulation. The relevant differential metabolites are shown in Table 1.

**Table 1:** Differential metabolites related to umbilical cord blood.

Number	Name	*p* value	Log2
HMDB0041513	Isopropyl apiosylglucoside	0.038146	−2.2246
HMDB0028893	Histidylproline	0.038842	−0.93135
HMDB0254199	Lumichrome	0.000885	−0.83041
HMDB0033019	7-Hydroxyterpineol 8-glucoside	0.02605	−0.79002
PubChem 71,367,139	1-Carboxy-L-prolylglycine	0.001828	−0.75223
HMDB0000619	Cholic acid	0.016129	−0.59337
HMDB0028683	Alanyl-aspartic	0.00154	−0.58209
HMDB0028752	Aspartyl-Glutamate	0.000723	−0.5117
HMDB0001173	5′-Methylthioadenosine	0.001014	−0.49611
HMDB0001844	Methylsuccinic acid	0.002747	−0.34886
HMDB0000730	Isobutyrylglycine	0.022298	−0.27564
HMDB0001434	3-Methoxytyrosine	0.047355	−0.27125
HMDB0011493	sn2 LysoPE(22:4)	0.02619	−0.26725
HMDB0003320	Indole-3-carboxylic acid	0.009991	−0.25279
HMDB0010724	3-Oxodecanoic acid	0.016325	−0.23795
HMDB0041815	6-Sulfatoxymelatonin	0.022372	−0.21514

**Figure 5:**
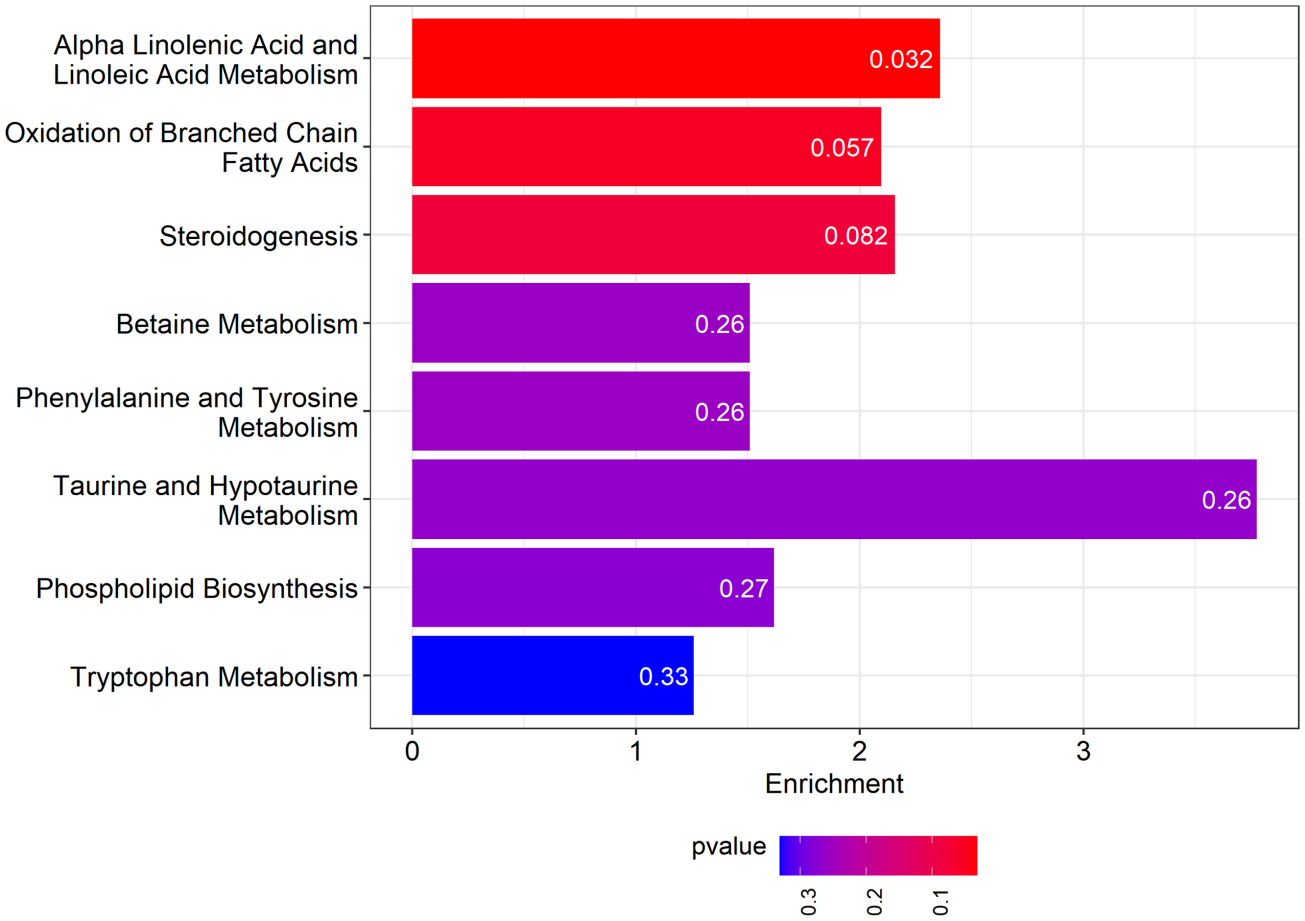
Metabolite set enrichment analysis.

Among the pathways enriched by the differential metabolites is Alpha-Linolenic Acid and Linoleic Acid Metabolism, for which the enrichment fold reaches 2.36. The results are presented in Figure 5.

## Discussion

Neonatal obesity is becoming increasingly prevalent worldwide and is one of the major health challenges nowadays. The causes of obesity remain unclear, but current research suggests that the occurrence of neonatal obesity partly originates from epigenetic modifications during the earliest stages of life, specifically the prenatal and perinatal periods. Maternal metabolism influences the metabolic programming of the fetus and newborn, thereby affecting the long-term metabolic health of the offspring (8, 9).

Metabolomics is an important tool for discovering new biomarkers and elucidating biochemical pathways. It can reveal the role of metabolites in the occurrence and development of diseases, providing significant value for studying disease mechanisms and prognosis (10). This study employs untargeted metabolomics analysis of maternal blood during pregnancy and umbilical cord blood at delivery, aiming to identify differential metabolites that can predict obesity, thereby enabling early intervention for neonatal obesity.

Through the analysis of maternal blood during pregnancy, differences in aspartyl-glutamate and alanyl-aspartate levels were observed between the obese and healthy groups. Both aspartyl-glutamate and alanyl-aspartate are derivatives of amino acids, and there is currently no research linking them to obesity. Aspartyl-glutamate is formed by the peptide bond between the *α*-amino group of asparagine and the carboxyl group of glutamate, potentially serving as an intermediate in certain metabolic pathways and participating in protein degradation or amino acid metabolism. Studies have shown that glutamate exhibits significant anti-obesity and insulin resistance-improving effects in high-fat diet-induced obese mice, primarily by regulating metabolic pathways (such as the TCA cycle) to promote energy expenditure and reduce fat accumulation. Additionally, glutamate can inhibit inflammatory signaling pathways (such as the NF-κB pathway), thereby reducing chronic inflammation and improving insulin resistance. Furthermore, as a neurotransmitter, glutamate may influence the function of the hypothalamic–pituitary axis to regulate appetite and energy expenditure. Alanyl-aspartate, on the other hand, is a dipeptide formed by the peptide bond between alanine and aspartate. Both alanine and aspartate are important metabolic amino acids, potentially related to energy metabolism, nitrogen metabolism, and neurotransmitter metabolism. Previous studies have shown that plasma levels of aspartate are significantly elevated in patients with obesity-related type 2 diabetes, and metabolites related to aspartate metabolism (such as asparagine and intermediates of the urea cycle) also undergo significant changes. Aspartate is a crucial intermediate in the TCA cycle, and its metabolic dysregulation may lead to abnormal energy metabolism. Aspartate also participates in the urea cycle, and its metabolic dysregulation may cause nitrogen metabolism abnormalities, further exacerbating metabolic burden. Currently, there is no research on the relationship between aspartyl-glutamate, alanyl-aspartate, and obesity. It is hypothesized that they may influence neonatal energy balance and fat storage by regulating the TCA cycle and amino acid metabolism.

In this study, the levels of aspartyl-glutamate and alanyl-aspartate in different stages of pregnancy and cord blood were analyzed. The results showed that the levels in the obese group were higher than those in the non-obese group, with significant differences observed in late pregnancy and cord blood. The levels of these metabolites were significantly elevated in maternal blood at different stages of pregnancy and in cord blood in the obese group. Although the roles of these two metabolites have not yet been confirmed, it is speculated that they may participate in the tricarboxylic acid (TCA) cycle and inflammatory stress responses to regulate glucose and lipid metabolism. Additionally, aspartyl-glutamate and alanyl-aspartate may serve as storage forms of aspartate and glutamate, releasing aspartate and glutamate through enzymatic reactions to participate in the TCA cycle, ultimately leading to abnormal energy metabolism. In the future, we will further validate these two metabolites, with the hope that early detection of metabolic abnormalities in newborns can be achieved through maternal blood during pregnancy or cord blood at birth, enabling timely postnatal interventions to reduce the incidence of neonatal obesity.

Further analysis of umbilical cord blood revealed differential metabolites such as isopropyl-D-aposide, carnosine, and 7-hydroxycitronellol-8-glucoside. Currently, there is a lack of research on the relationship between these metabolites and obesity. Isopropyl-D-aposide, a glycoside compound, is hypothesized to potentially reduce the risk of obesity by alleviating oxidative stress and chronic inflammation, thereby improving insulin sensitivity and energy metabolism. Additionally, this compound may indirectly influence the occurrence of obesity by modulating the composition of gut microbiota, affecting energy absorption and metabolism.

Regarding carnosine, there are limited reports in the literature. Previous studies have shown that histidyl-proline diketopiperazine (His-Pro DKP), a dipeptide derivative formed by the cyclization of histidine and proline, plays an important role in controlling appetite and regulating insulin and glucagon (11). It is speculated that carnosine may exert its effects on glucose metabolism through further cyclization in the body, ultimately contributing to neonatal obesity.

7-Hydroxycitronellol-8-glucoside is a compound formed by the glycosidic bond between 7-hydroxyterpineol and glucose. 7-Hydroxyterpineol exhibits antioxidant activity, suggesting that it may protect cells from oxidative damage by scavenging free radicals. Furthermore, 7-hydroxyterpineol possesses anti-inflammatory properties, which may reduce the risk of obesity by mitigating inflammatory responses.

Through further analysis of cord blood, we identified more differential metabolites. Although current research on these metabolites is relatively limited and their specific roles in neonatal obesity remain unclear, the diversity of metabolites provides more ideas and research directions for exploring biomarkers that can predict neonatal obesity in the future. We look forward to using targeted metabolomics to validate these differential metabolites, identify those involved in glucose and lipid metabolism and contributing to neonatal obesity, and ultimately apply them for the prediction and intervention of neonatal obesity.

The strength of this study lies in its use of a prospective longitudinal cohort design, which enables detailed data collection at different time points throughout pregnancy as well as comprehensive documentation of pregnancy-related conditions. Additionally, follow-up of newborns after birth provides robust data support for the research. However, as the study only tracked newborns up to 6 months of age, whether abnormalities in glucose and lipid metabolism will develop further during growth, or whether the obese state will persist with development, requires further long-term follow-up. It takes approximately 1 year to obtain specimens from each participant after enrollment. Although our single center handles a very high delivery volume, participants are enrolled during early pregnancy, making it impossible to predict in advance who will subsequently develop glucose-lipid metabolic disorders or deliver overweight newborns. As a result, many cases are excluded and the final sample size is relatively small. We will continue to accumulate additional cases and attempt to launch a multicenter enrollment to gather a larger cohort, which will allow us to further validate our findings in future analyses. In addition, the specific mechanisms of differential metabolites in glucose and lipid metabolism that we have identified need to be further validated through experimental design. Furthermore, in this study, umbilical venous blood was extracted for analysis, which can better reflect the placental-to-fetal circulation and may carry a stronger maternal signature than arterial cord blood. Although this homogeneous compartment mitigates variation introduced by mixed sampling, it limits direct inference on purely fetal metabolic status. Future studies with paired arterial–venous sampling will be valuable for disentangling maternal versus fetal metabolic contributions.

This study analyzed maternal blood during early, mid, and late pregnancy, as well as cord blood, aiming to predict obesity during the embryonic stage of newborns and enable early intervention in utero. Although no differential metabolites were identified in early pregnancy at this stage, the findings provide new ideas and directions for future research.

This study aims to identify biomarkers that facilitate the early detection of neonatal obesity by analyzing maternal blood during pregnancy and umbilical cord blood at birth. The differentially identified biomarkers currently screened are primarily dipeptides related to aspartic acid and glutamic acid, which are involved in various biological processes such as gut microbiota regulation, glucose metabolism, and lipid metabolism. However, the specific mechanisms by which these biomarkers participate in and influence these processes remain to be further investigated. In subsequent research, we plan to validate these biomarkers using targeted omics approaches and further analyze the related pathways to explore sensitive molecular biomarkers capable of predicting neonatal obesity.

## Data Availability

The data that support the findings of this study are available from the corresponding author upon reasonable request.
